# On Bougies of Gelatine

**Published:** 1876-12

**Authors:** 


### EXCERPTA.

---

On Bougies of Gelatine.—These are much used in Vienna; a correspondent of the *Medical Examiner* says their use was first introduced at the Laryngoscopic Clinic, and it has been attended with great success. The bougies used resemble those already employed in urethral diseases; they are something over three inches in length, and from one-eighth to one-fourth of an inch in diameter, and are pointed at one end, so as to be more easily introduced. The drugs most commonly applied in this way are the astringents, as alum, sulphate of copper, rhatany, and carbolic acid. Hitherto the treatment of nasal disease has been confined to injections of tepid water and solutions of different drugs, and applications of caustics to the nasal mucous membrane by means of a *porte-caustique*, the latter of which methods causes intense pain when the mucous membrane is swollen and the meatus is narrow. Further, cauterization cannot be employed sufficiently often. The introduction of the nasal bougie, on the contrary, is not at all painful; the elastic body adapts itself to every irregularity in the nasal cavity, passes very easily through the narrowest parts of the meatus, and dilates them by a gentle pressure.

These bougies have been used in cases of coryza, ozæna, and with great success in cases of extensive swelling of the nasal mucous membrane and of the turbinated bones. If there is total obstruction of the meatus, and air cannot be drawn through the nostril, the introduction of the first bougie often effects a marvelous improvement. In cases of ozæna, sulphate of copper and carbolic acid are the most useful agents, but where there is extensive swelling and relaxation of the mucous membrane, the tincture of rhatany is to be recommended. Sulphate of zinc is not much used, for, according to Stork's experiments, solutions of this drug, even when only *injected* into the nose, destroy the power of smell.

There is no difficulty in introducing the bougie. It is advisable to give it a rotary as well as an onward motion during introduction. Even in the most obstructed meatus, it is possible to introduce the bougie completely, and in any direction. Afterward the nostril is plugged with lint to prevent the liquefied gelatine from escaping by any other orifice than the posterior nares. When there is much secretion present the bougie may liquefy in three-quarters of an hour, but it usually takes three hours. It causes no unpleasant sensation while in the nose, and it is useful not only in applying medicaments to the mucous membrane, but in keeping the meatus dilated.

